# 37. THE GUT MICROBIOME: A KEY REGULATOR OF NEURODEVELOPMENT AND BEHAVIOUR

**DOI:** 10.1093/schbul/sby014.152

**Published:** 2018-04-01

**Authors:** John Cryan

**Affiliations:** University College Cork

## Abstract

**Overall Abstract:** The brain-gut-microbiota axis is emerging as a research area of increasing interest for those investigating the biological and physiological basis of neurodevelopmental, age-related and neurodegenerative disorders. The routes of communication between the gut and brain include the vagus nerve, the immune system, tryptophan metabolism, via the enteric nervous system or by way of microbial metabolites such as short chain fatty acids. These mechanisms also impinge on neuroendocrine function at multiple levels. Studies in animal models have been key in delineating that neurodevelopment and the programming of an appropriate stress response is dependent on the microbiota. Developmentally, a variety of factors can impact the microbiota in early life including mode of birth delivery, antibiotic exposure, mode of nutritional provision, infection, stress as well as host genetics. At the other extreme of life, individuals who age with considerable ill health tend to show narrowing in microbial diversity. Stress can significantly impact the microbiota-gut-brain axis at all stages across the lifespan. Recently, the gut microbiota has been implicated in a variety of conditions including obesity, autism, schizophrenia and Parkinson’s disease. Moreover, animal models have been key in linking the regulation of fundamental brain processes ranging from adult hippocampal neurogenesis to myelination to microglia activation by the microbiome. Finally, studies examining the translation of these effects from animals to humans are currently ongoing. Further studies will focus on understanding the mechanisms underlying such brain effects and developing nutritional and microbial-based intervention strategies.

